# Functional pathways regulated by microRNA networks in CD8 T‐cell aging

**DOI:** 10.1111/acel.12879

**Published:** 2018-11-28

**Authors:** Claire E. Gustafson, Mary M. Cavanagh, Jun Jin, Cornelia M. Weyand, Jörg J. Goronzy

**Affiliations:** ^1^ Division of Immunology and Rheumatology, Department of Medicine Stanford University School of Medicine Stanford California; ^2^ Department of Medicine Palo Alto Veterans Administration Healthcare System Palo Alto California

**Keywords:** cellular homeostasis, FOXO1, IL‐7 receptor, immunosenescence, posttranscriptional regulation, TNF‐alpha

## Abstract

One of the most prominent immunological changes during human aging is the alteration in CD8 T‐cell subset distribution, predominated by a loss of naïve CD8 T cells. The molecular mechanisms that contribute to the loss of naïve CD8 T‐cells during aging remain unclear. Considering that many CD8 T‐cell functions are influenced by microRNAs (miRNAs), we explored miRNA expression profiling to identify novel dysfunctions that contribute to naïve CD8 T‐cell loss during aging. Here, we describe age‐dependent miRNA expression changes in naïve, central memory, and effector memory CD8 T‐cell subsets. Changes in old naïve CD8 T‐cells partially resembled those driven by an underlying shift in cellular differentiation toward a young central memory phenotype. Pathways enriched for targets of age‐dependent miRNAs included FOXO1, NF‐κB, and PI3K‐AKT signaling. Transcriptome analysis of old naïve CD8 T‐cells yielded corresponding patterns that correlated to those seen with reduced FOXO1 or altered NF‐κB activities. Of particular interest, IL‐7R expression, controlled by FOXO1 signaling, declines on naïve CD8 T cells with age and directly correlates with the frequencies of naïve CD8 T cells. Thus, age‐associated changes in miRNA networks may ultimately contribute to the failure in CD8 T‐cell homeostasis exemplified by the loss in naïve cells.

## 1 INTRODUCTION

Human aging is commonly characterized by increased susceptibility to infections (e.g., influenza, *Streptococcus pneumonia*), which account for 2.6% of deaths in adults 65 years or older in the U.S. This age‐related susceptibility to infections has been linked with the reduced ability of the aging immune system to mount effective adaptive immune responses, which includes impaired T‐cell responses to viral pathogens and vaccination (Goronzy & Weyand, 2017; Nikolich‐Zugich, 2018). The CD8 T‐cell compartment is particularly affected by age, with a striking and highly reproducible decline in the number of naïve CD8 T cells (Czesnikiewicz‐Guzik et al., 2008; Wertheimer et al., 2014). The naïve CD8 T‐cell compartment from older individuals not only decreases in numbers but also undergoes significant repertoire contraction (Qi et al., 2014) and both transcriptionally and epigenetically displays more memory‐like characteristics than that of young adults (Moskowitz et al., 2017; Ucar et al., 2017).

Previous studies have identified miRNAs as important regulators of effector CD8 T‐cell responses and of memory differentiation (Wu et al., 2012; Zhang & Bevan, 2010). Indeed, global knockouts of miRNAs caused by deletions in the miRNA‐processing pathway affect both effector function and proliferative capacity of CD8 T‐cells after activation (Trifari et al., 2013; Zhang & Bevan, 2010). Targeted approaches have also linked specific miRNAs with CD8 T‐cell transition from effector to memory phenotype (Khan, Penny, Yuzefpolskiy, Sarkar, & Kalia, 2013; Smith, Wissink, Grimson, & Rudd, 2015), with the ability of CD8 T cells to clear viral infections (Wang et al., 2013), with the accumulation of terminally differentiated effector memory CD8 T cells (Brunner et al., 2012) and with dysfunctional T‐cell receptor signaling in the elderly (Li et al., 2012). During early development, miRNA expression changes in neonatal CD8 T‐cells associate with inability of neonatal mice to develop memory T cells (Wissink, Smith, Spektor, Rudd, & Grimson, 2015). Moreover, multiple age‐associated miRNA differences have been found in terminally differentiated effector CD8 T‐cells from humans (Hackl et al., 2010; Teteloshvili et al., 2015). However, whether naïve CD8 T‐cell subsets display altered miRNA expression during human aging and whether age‐associated miRNAs influence the functionality of these CD8 T cells has not been investigated.

In this study, we examined the age‐associated heterogeneity of miRNA expression in highly purified naïve, central memory, and effector memory CD8 T‐cell subsets and the functional outcomes of these changes in the naïve CD8 T‐cell compartment. We identified multiple subset‐specific miRNA expression changes with age. Notably, age‐dependent miRNA alterations in the naïve CD8 T‐cell compartment partially reflected that of cellular differentiation toward a central memory phenotype. Moreover, global network analysis of these age‐dependent miRNA changes in naïve CD8 T‐cells implicated a potential role for the FOXO1 signaling pathway in defective naïve CD8 T‐cell homeostasis during human aging.

## 2 RESULTS

### 2.1 Age‐dependent alterations in miRNA expression across CD8 T‐cell subsets

We initially addressed whether aging caused miRNA changes in naïve and memory CD8 T‐cell subsets in a cohort of young (<30 years, *n* = 15) and older (≥65 years, *n* = 9) adults. The median age of the young cohort was 21 years (range: 16–28). The median age of the older cohort was 70 years (range: 65–82). There was no statistical difference in the distribution of gender (*p* = 0.403) between the two cohorts (gender: 33% female in young and 56% female in older). As expected, there was a trend for higher CMV positivity in the older cohort (*p* = 0.09; 27% CMV positive in young vs. 67% in old).

**Figure 1:**
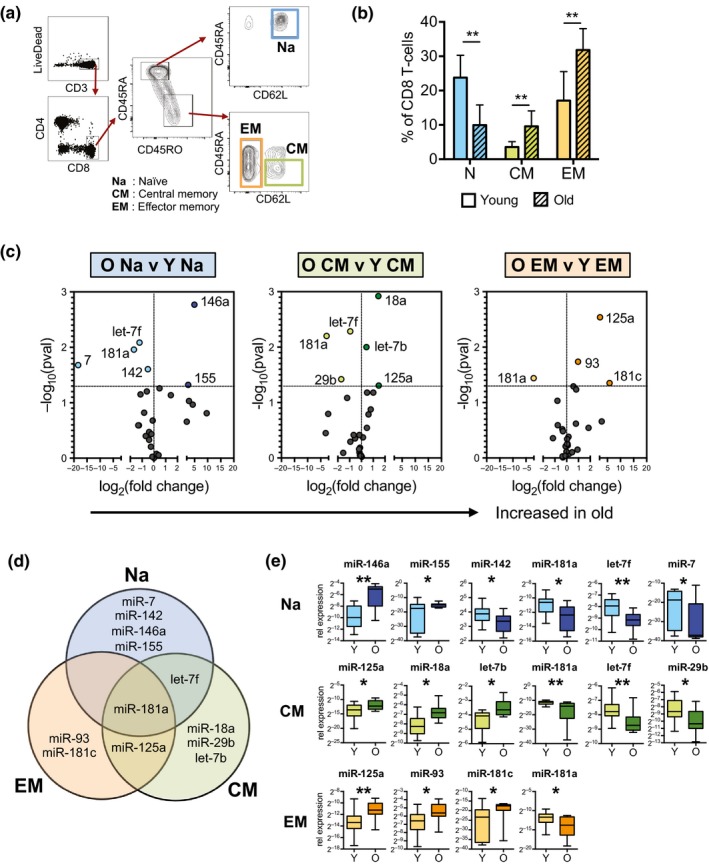
Age‐dependent miRNA expression changes in CD8 T‐cell subsets. (a) Cell sorting strategy for naïve (Na), central memory (CM), and effector memory (EM) subsets pregated on single lymphocytes by forward and side scatter. (b) Frequencies of naïve, CM, and EM subsets within the CD8 compartment of young (*n* = 12) and older (*n* = 6) cohort. (c) Volcano plots of miRNA expression differences between young (Y, <30 years, *n* = 15) and old (O, ≥65 years, *n* = 9) CD8 T‐cell subsets. Upper right quadrant indicates miRNAs that are increased in older individuals. (d) Overlap between age‐dependent miRNA changes from each CD8 T‐cell subset. (e) Expression of age‐dependent miRNA changes in naïve, CM, and EM CD8 T‐cell subsets by age. *p*‐Values were determined using Mann–Whitney test (*p < 0.05, **p < 0.01)

As miRNA studies on highly purified aged CD8 T‐cell subsets are limited, we sorted naïve, central memory, and effector memory subsets from peripheral blood of these healthy adults (Figure 1a). Consistent with previous studies, we found reduced frequencies of naïve CD8 T cells and increased frequencies of the memory CD8 populations in older individuals (Figure 1b). Equal numbers of naïve and memory cells were simultaneously screened for expression of 34 preselected, immune‐related miRNAs by multiplex microfluidic qPCR. We then compared miRNA expression levels between the three cell subsets in young and older individuals. miRNA expression levels relative to RNU48 are listed in Supporting Information Table S1. We found age‐dependent miRNA differences in all three subsets (Figure 1c). Notably, most of the miRNA differences were unique to the individual subset, with a majority of these age‐dependent miRNA changes found within the naïve compartment (Figure 1d). One out of the 12 age‐dependent changes was found in all three subsets; this change being a reduction of miR‐181a in older individuals. miRNAs specific to naïve CD8 T‐cells included miR‐146a, miR‐155, miR‐142, and miR‐7. miR‐146a (increased) and let‐7f (decreased) were the most significantly altered miRNAs in the naïve population (Figure 1e). miR‐155 and miR‐7 were both expressed close to the level of detection and were not detected in some individuals giving the appearance of a bimodal distribution. Naïve and CM CD8 compartments shared a significant decrease in let‐7f expression with age. CM and EM compartments shared increased miR‐125a expression. Naïve and EM compartments shared no unique age‐related miRNA changes. Thus, we identified multiple miRNA expression changes in CD8 T cells that are subset‐ and age‐dependent.

### 2.2 CMV status does not affect miRNA expression in CD8 T‐cell subsets

**Figure 2:**
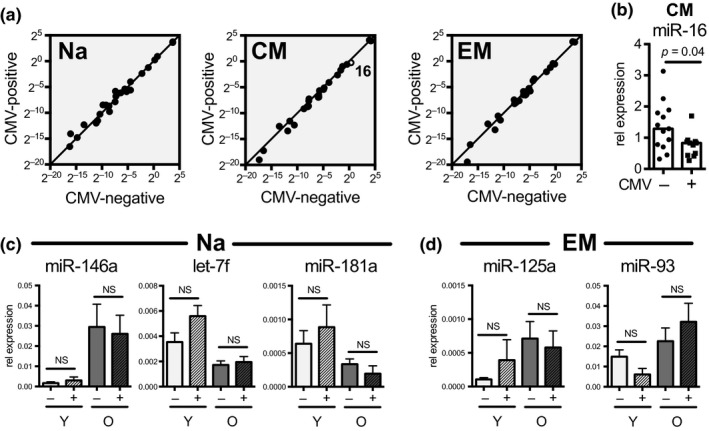
Effect of CMV infection on miRNA expression in CD8 T‐cell subsets. (a) Correlation between median miRNA expression in CMV‐negative (*n* = 13) and CMV‐positive (*n* = 10) individuals, stratified for CD8 subsets. Bold numbers indicate significantly changed miRNAs, as determined by Mann–Whitney test. (b) miR‐16 expression in CM CD8 T‐cells based on CMV status. (c) Expression of miR‐146a, let‐7f, and miR‐181a in Na CD8 T‐cells separated by CMV status and age. (d) Relative expression levels of miR‐125a and miR‐93 in effector memory EM CD8 T‐cell separated by Y CMV−, Y CMV+, O CMV−, and O CMV+ (*n* = 10, 4, 3, and 6, respectively). Significance was determined by Mann–Whitney test. NS: not significant

Many molecular and cellular changes in CD8 T cells during aging have been linked with CMV infection. In our cohort, 26.7% (4 out of 15) of young donors and 66.7% (6 out of 9) of old donors had a positive CMV serology. Therefore, we investigated whether underlying CMV infection influenced miRNA expression in CD8 T‐cell subsets and contributed to age‐dependent miRNA changes. However, no significant differences in miRNA expression were found between CMV‐negative and CMV‐positive individuals in the naïve and effector memory compartments (Figure 2a). The central memory compartment showed slightly decreased miR‐16 expression in CMV‐positive individuals, but this miRNA did not overlap with differences observed in aging (Figure 2b). When further separated by age and CMV status, we again find no differences in miRNA expression, including miR‐146a, let‐7f, and miR‐181a in naïve CD8 T cells (Figure 2c). Effector memory cells also showed little miRNA differences with CMV and age separation, including miR‐125a and miR‐93 that significantly change with age in EM populations (Figure 2d). Although these analyses have small sample numbers, power was sufficient to detect differences of about two standard deviations (*SD*). Specifically, for comparing 4 young CMV+ with 10 young CMV−, we have 80% power for detecting a difference of 1.86 *SD* based on two‐sided *t* test. Similarly, for comparing three old CMV− with six old CMV+, we have 80% power for detecting a difference of 2.38 *SD*. One young donor was removed from the analysis, as there was no CMV status available. Overall, these data suggest that CMV infection does not significantly influence age‐dependent miRNA changes in naïve, central memory, or effector memory CD8 T‐cell subsets.

### 2.3 Detection of differentiation‐dependent miRNA signature in CD8 T‐cells

**Figure 3:**
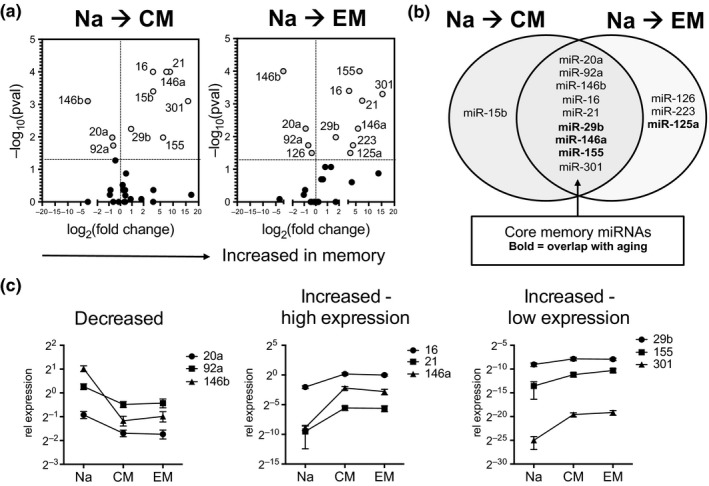
Differentiation‐dependent miRNA expression in CD8 T cells from young adults. (a) Volcano plots of miRNA expression differences comparing naïve (Na) and central memory (CM) or effector memory (EM) CD8 T‐cell subsets from young adults (*n* = 15 per subset). Fold change indicates median expression of memory divided by median expression of naïve population. (b) Overlap between miRNA changes in naïve vs. CM and naïve vs. EM subsets in young adults. (c) Relative expression values of core memory miRNAs separated by expression levels and directionality of change. Graph shows mean ± *SEM*

One possible driver of age‐dependent changes is cellular differentiation. To determine differentiation‐driven miRNA changes, we first analyzed miRNA differences between naïve and memory populations in young adults. Analysis of our select panel of miRNAs identified multiple miRNA expression changes with differentiation (13 miRNAs, 32.5%), with a majority of miRNAs having increased expression in the memory populations (Figure 3a). Central and effector memory populations displayed no distinct miRNA expression differences. Comparing the expression differences between naïve and memory populations in young adults, we identified a group of nine miRNAs commonly changed during differentiation to central as well as effector memory cells; we termed these miRNAs as core memory miRNAs (Figure 3b). Core memory miRNAs included miR‐120a, miR‐92a, and miR‐146b, which are down‐regulated with differentiation, and miR‐16, miR‐21, miR‐29b, miR‐146a, miR‐155, and miR‐301, which are up‐regulated with differentiation, albeit at different levels of expression (Figure 3c). Previous studies on miRNA profiles during CD8 T‐cell differentiation also identified that miR‐21, miR‐146a, and miR‐155 increased in the memory population with a coinciding decrease in miR‐92 and miR‐146b in humans (Salaun et al., 2011), suggesting these miRNAs are highly robust markers of human naïve vs. memory CD8 T cells.

### 2.4 Partial differentiation of aged naïve CD8 T‐cells influences miRNA expression

**Figure 4:**
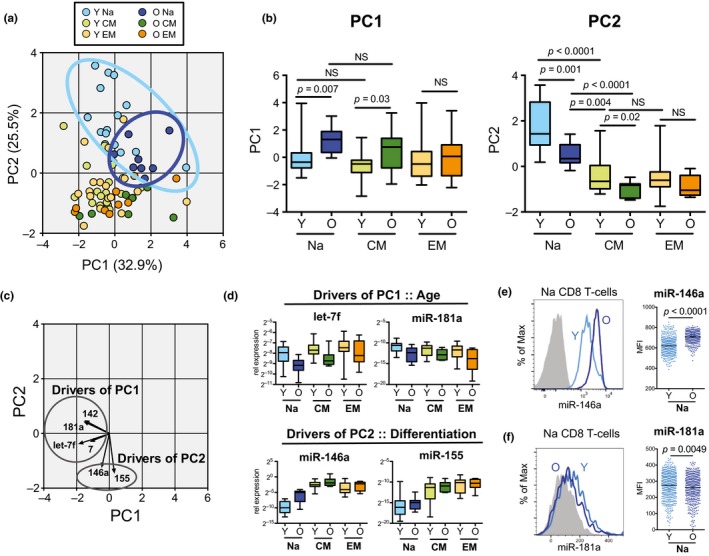
Relationship between age‐ and differentiation‐dependent miRNA changes in naïve CD8 T‐cells. (a) Principal component analysis (PCA) of the expression of the six age‐dependent miRNAs in naïve CD8 T‐cells across all young (Y) and old (O) CD8 T‐cell subsets. (b) Box plots of PC1 and PC2 variance for each subset by age. *p*‐Values were determined using one‐way ANOVA. (c) Vector plot of the individual contribution of each age‐dependent miRNA to the PCA. Longer arrows indicate a stronger contribution. (d) Box plots of the relative expression of miRNAs involved in PC1 (miR‐181a and let‐7f) and PC2 (miR‐146a and miR‐155) from young (*n* = 15) and old (*n* = 9) individuals. (e) miR‐146a and (f) miR‐181a expression in naïve CD8 T cell from young and old individuals determined by flow cytometry. Dot plots show single cell expression levels for 500 naive CD8 T cells per individual. Histogram and dot plot are representative of three independent experiments. *p*‐Values were determined by Mann–Whitney test

We next investigated the relationship between the six age‐dependent miRNAs in naïve CD8 T cells (miR‐146a, miR‐155, miR‐181a, miR‐142, miR‐7, and let‐7f) and their expression during cellular differentiation, utilizing principal component analysis (PCA; Figure 4a). We found that PC1 (32.9% of variation in dataset) was driven by age‐dependent changes, seen by differences in expression between young and old (Young Na vs. Old Na [*p* = 0.007]; Young CM vs. Old CM [*p* = 0.03]) but not between cell subsets (i.e., Na vs. CM; Figure 4b). PC2 (25.5% of the variation) showed differences between young and old cells as well as between cell subsets, suggesting a role for differentiation in this secondary variation. No age difference was observed between young and old effector memory populations, consistent with these cells being closer to end differentiation than naïve and central memory T cells.

To understand how the individual miRNAs are driving the distribution across PC1 and PC2, we overlaid their corresponding vectors onto the PCA plot. We found that PC1 variation was driven mostly by miR‐7, miR‐142, miR‐181a and let‐7f, whereas PC2 was driven by miR‐146a and miR‐155 (Figure 4c). Individual expression plots of these miRNAs confirmed strong relationships with age for miR‐181a and let‐7f whereas miR‐146a and miR‐155 were influenced by cellular subset (Figure 4d). Notably, expression of miR‐146a showed a stepwise increase from young naïve to old naïve to young central memory.

It is possible that the increase of miR‐146a in aged naïve CD8 T cells could be caused by stem‐like memory cells or contamination with other memory cells that are phenotypically difficult to distinguish from naïve T cells (Akondy et al., 2017; Fuertes Marraco et al., 2015; Gattinoni et al., 2011). Thus, we next interrogated single cell expression of miR‐146a in young and old naïve CD8 T‐cells utilizing flow cytometry in combination with in situ hybridization for miRNA detection. We found that expression of miR‐146a followed a unimodal distribution with the entire population of aged naïve CD8 cells shifted toward higher miR‐146a (Figure 4e), demonstrating that increased miR‐146a expression in aged naïve CD8 T cells was not driven solely by a naïve‐like memory subpopulation. We also find an overall decrease in miR‐181a in aged naïve CD8 cells using the same detection method (Figure 4f). Therefore, the expression pattern of these six miRNAs in naïve CD8 T cells is unique to aging and not driven by contamination of “virtual” naïve cells, such as stem cell‐like memory. These data also reveal that differentiation of the naïve compartment partially, but not solely, accounts for the miRNA expression profile in old naïve CD8 T cells.

### 2.5 Age‐dependent miRNAs in naïve CD8 T‐cells predict FOXO1 signaling defect

**Figure 5:**
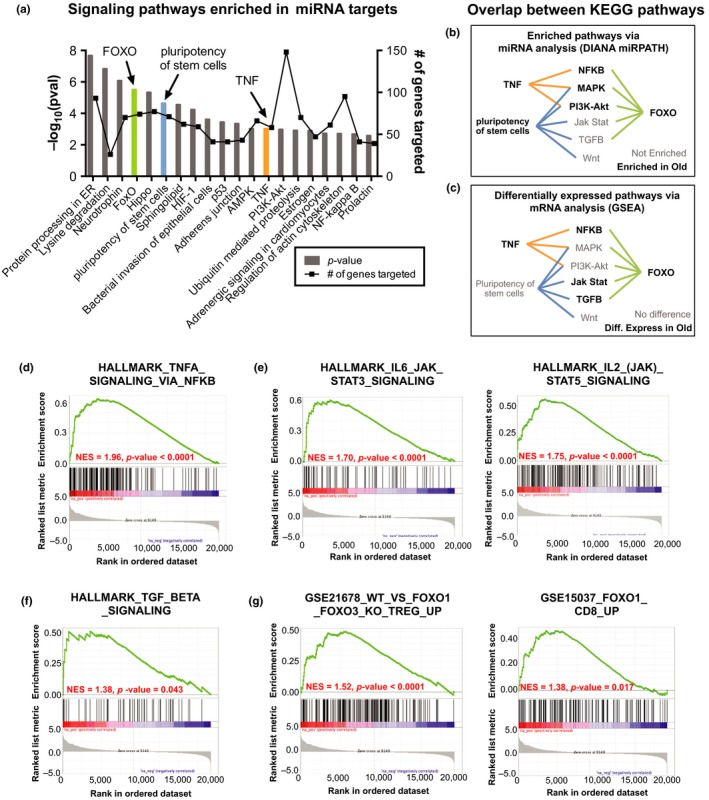
Targeted pathways of the age‐dependent miRNA network in naïve CD8 T cells. (a) Top 20 enriched signaling pathways from DIANA miRPATH analysis using the six age‐dependent miRNAs in naïve CD8 T cells (miR‐146a, miR‐155, let‐7f, miR‐181, miR‐7, and miR‐142). Left Y axis is the enrichment *p*‐value for pathway from DIANA miRPATH analysis. Right Y axis is the number of genes targeted within the pathway. (b, c) Pathway overlap between TNF, FOXO, and pluripotency in stem cell pathways highlighting pathways that are (b) enriched for targets of age‐related miRNAs or (c) differentially expressed in RNAseq data from old compared with young naïve CD8 T cells. Bold indicates significantly enriched pathways. (d–g) Gene set enrichment analysis (GSEA) analyzes comparing mRNA changes induced during naïve CD8 T‐cell aging (young compared with old naïve CD8 T cells) with that of hallmark pathways for (d) TNFα signaling, (e) JAK‐STAT signaling, and (f) TGFβ signaling. (g) GSEA analyzes comparing mRNA changes induced during naïve CD8 T‐cell aging with that of experimental datasets from FOXO1/FOXO3 or FOXO1 knockout T‐cells

miRNAs primarily function as posttranscriptional regulators, by binding the mRNA and inducing mRNA degradation or preventing protein translation. However, miRNAs have hundreds of different mRNA targets and determining the function of multiple miRNA changes at a cellular level is extremely challenging. As a result, studies are often limited to one‐on‐one miRNA and mRNA interactions. Here, we utilized a computational tool (i.e., DIANA mirPATH; see Section 4) that provides a global picture of cellular function based on miRNA profiles, by integrating all known targets of input miRNAs and providing an enrichment score for a pathway based on the number of miRNA targets genes present. From this analysis, we found multiple signaling pathways enriched in experimentally validated targets for age‐dependent miRNAs in naïve CD8 T cells (Figure 5a). Top KEGG pathways included protein processing in the endoplasmic reticulum (*p* = 2.15E‐8 with 93 targets), lysine degradation (*p* = 1.48E‐7 with 26 gene targets), neurotrophin signaling (*p* = 8.36E‐7 with 70 targets), FOXO signaling (*p* = 3.06E‐6 with 74 targets), and pluripotency of stem cells (*p* = 2.26E‐5 with 71 target genes). The PI3K‐AKT signaling pathway (*p* = 0.001 with 148 targets) demonstrated the highest number of genes targeted by age‐dependent miRNAs. Interestingly, TNFα signaling (*p* = 0.0009 with 58 gene targets) has been previously identified as a signaling defect in aging naïve T cells (Gupta & Gollapudi, 2006).

KEGG pathways usually integrate multiple upstream and downstream signaling molecules; thus, there is often a high level of redundancy in pathway assignments. Therefore, we investigated the functional overlap between three select pathways: FOXO, pluripotency in stem cells, and TNFα signaling. These pathways demonstrated multiple mRNA targets for all six age‐related miRNAs with robust targeting of multiple genes within each pathway (Supporting Information Figure S1). Moreover, we found a high overlap between individual signaling pathways contained within these three pathways (Figure 5b). For example, all three pathways contained MAPK and PI3K‐AKT signaling pathway components, which are also significant pathways targeted by age‐related miRNAs (MAPK *p* = 0.018 with 107 gene targets; Figure 5a).

To determine whether miRNA prediction of pathway dysfunction can be validated at the transcriptome level, we compared transcriptome differences in young and old naïve CD8 T cells from humans with functionally informative “hallmark” mRNA expression datasets via gene set enrichment analysis (GSEA). Details on these datasets are available in Section 4. Similar to DIANA miRPATH analysis, we found significant enrichment in gene profiles for TNFα signaling via NF‐κB and FOXO1 signaling (Figure 5b). Of note, three out of five pathways with differential gene expression in old naïve CD8 T cells were linked with FOXO1. These pathways included NF‐κB, JAK‐STAT, and TGF‐β signaling. All three of these pathways demonstrated gene expression profiles consistent with altered signaling in old naïve CD8 T cells (Figure 5d–f). We also found that old naïve CD8 T‐cells displayed a similar gene expression profile as that of mouse FOXO1 knockout cells (Figure 5g), indicating that old naïve CD8 T cells have reduced FOXO1 signaling. The decrease in FOXO1 signaling in old naïve CD8 T cells was not due to altered FOXO1 protein expression in old naïve CD8 T cells (Supporting Information Figure S2a).

### 2.6 FOXO1 signaling is reduced in old naïve CD8 T‐cells

**Figure 6:**
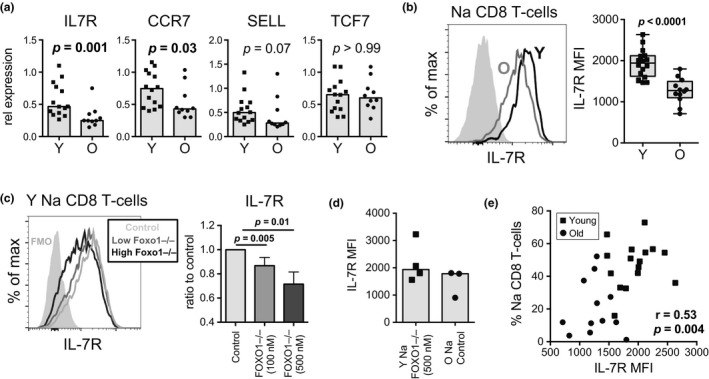
FOXO1 signaling and naïve CD8 T‐cell aging. (a) mRNA expression of selected FOXO1 target genes (IL7R, CCR7, SELL, TCF7) from naïve CD8 T‐cells isolated from young (Y; *n* = 14) and older (O; *n* = 10) individuals; comparison by Mann–Whitney test. (b) IL‐7R protein expression on resting naïve CD8 T cells from young (*n* = 16) and older (*n* = 12) individuals; comparison by Mann–Whitney test. (c) IL‐7R protein expression after 24 hr of FOXO1 inhibition using AS1842856 (100 or 500 nM as indicated) in young (*n* = 4) naïve CD8 T cells. Histogram is representative of three independent experiments. *p*‐Values were determined by paired *t* tests. (d) Comparison of IL‐7R expression on FOXO1 inhibited young naïve CD8 T cells and resting old naïve CD8 T cells. (e) Pearson's correlation between baseline expression of IL‐7R by naïve CD8 T cells and the frequencies of peripheral naïve CD8 T cells in young (*n* = 16; squares) and older (*n* = 12; circles) adults

To establish the functional activity of FOXO1 in naïve CD8 T cells, we compared gene expression of select FOXO1 target genes (IL7R, CCR7, SELL, TCF7) in a follow‐up cohort of young and older individuals by qPCR. IL7R and CCR7 mRNA expression were significantly decreased in old naïve CD8 T cells (Figure 6a). SELL trended lower in older individuals. TCF7 did not have altered expression between young and old, consistent with a recently published study showing that FOXO1 induces TCF7 expression in memory, but not in naïve, CD8 T cells (Delpoux, Lai, Hedrick, & Doedens, 2017). Consistent with significantly decreased IL7R mRNA expression, IL‐7R surface protein expression on old naïve CD8 T cells was lower than that of young (Figure 6b). To demonstrate a direct link between FOXO1 activity and IL‐7R expression, we inhibited FOXO1 activity in naïve CD8 T‐cells in vitro. Inhibition of FOXO1 function in young naïve CD8 T‐cells decreased IL‐7R expression (Figure 6c), demonstrating that FOXO1 function can directly affect IL‐7R expression levels in human naïve CD8 T cells. Moreover, high dose FOXO1 inhibition reduced IL‐7R expression on young naïve CD8 T cells to levels similar to that of old naïve CD8 T‐cells (Figure 6d). CCR7 was not affected by FOXO1 inhibition nor did naïve CD8 T cells from older individuals display decreased surface expression of this receptor (Supporting Information Figure S2b,c), indicating that CCR7 may not be a direct transcriptional target of FOXO1 in human naïve CD8 T cells.

At a functional level, IL‐7R expression by naïve CD8 T cells is important for cell survival and homeostatic proliferation. Aging is associated with decreased frequencies of naïve CD8 T cells (Figure 1b); thus, this decrease in frequency may be connected with the loss of IL‐7R. Consistently, we found that the basal level of IL‐7R expression on naïve CD8 T‐cells positively correlated with frequency of peripheral naïve CD8 T cells (Figure 6e). Conversely, no correlation with CCR7 expression was observed (Supporting Information Figure S2d). Together, these data suggest that the FOXO1‐IL7R axis may play a role in the loss of naïve CD8 T‐cells during aging.

## 3 DISCUSSION

Human immune aging is characterized by a significant and disproportional loss in the naïve CD8 compartment. Mounting evidence suggests that the loss of naïve CD8 T‐cells is driven by cell‐intrinsic changes. Here, we show that the expression of multiple miRNAs is altered within peripheral CD8 T‐cell subsets from older individuals, with changes found in naïve CD8 T‐cells partially driven by cellular differentiation but not by underlying CMV infection or stem cell memory expansion. Moreover, we demonstrate that miRNA network analysis can be used to predict and identify functional changes in immune cell subsets during aging. Indeed, we identified the FOXO1 pathway as a potential target of the age‐dependent miRNA network in naïve CD8 T cells and propose that reduced FOXO1 activity may contribute to declining naïve CD8 T‐cell homeostasis observed during aging. As miRNAs are important, posttranscriptional regulators of protein expression, these findings reveal a new layer of cell‐intrinsic dysfunction in T‐cell aging that cannot be detected by transcriptional or epigenetic profiling alone. These data also suggest that miRNAs, or their functional targets, could be promising avenues to pursue in order to improve long‐term CD8 T‐cell homeostasis and boost response to pathogenic infection during aging.

During human aging, the thymus, which generates new T cells, involutes. Thus, naïve CD8 T cells are maintained in older individuals by homeostatic mechanisms. In this context, age‐related miRNA changes may be driven by factors mediating long‐term homeostatic maintenance. The two main factors that mediate T‐cell homeostasis are (a) low‐affinity TCR interactions (with a self‐antigen) and (b) cytokine signaling via IL‐7 and IL‐15. Notably, IL‐15 can induce miR‐146a expression by naïve CD8 T cells (Sheppard et al., 2014). This expression of miR‐146a is alternatively inhibited by IL‐7, suggesting that age‐related increases in miR‐146a (Figure 1a) could be, at least partially, mediated by altered signaling via homeostatic cytokines. High‐affinity TCR engagement also induces expression of many miRNAs including miR‐146a and miR‐155 (Teteloshvili et al., 2015); however, the effects of low‐affinity TCR stimulation and the interplay between cytokines and TCR signaling in regulating miRNA expression are currently unknown.

Our study presented here revealed that old naïve CD8 T‐cells also exhibit miRNA expression patterns partially related to cell differentiation. The idea that naïve T‐cells undergo partial differentiation during aging is a relatively new idea and somewhat controversial, because, as we age, there is an increase in a naïve‐like memory population (Eberlein et al., 2016; Pulko et al., 2016) that expresses basic phenotypic surface receptors highly similar to that of naïve CD8 T cells (e.g., CCR7, CD45RA, CD62L; Fuertes Marraco et al., 2015; Gattinoni et al., 2011). This memory population therefore could be contributing to the differentiation‐related changes in miRNAs (and other molecular changes) found within the aging naive compartment. However, we demonstrate this is an unlikely sole explanation because the shift in miRNA expression, in particular miR‐146a, was a global phenomenon across the entire aged naïve CD8 T‐cell compartment (Figure 4). Moreover, we find little overlap between age‐dependent and differentiation‐dependent miRNAs in CD8 T‐cells, suggesting that although differentiation may contribute to some aspects of aging within the global T‐cell compartment, it does not account for all changes.

Interestingly, a population of “virtual memory” cells has recently been found in mice and shown to increase with age (Nikolich‐Zugich, 2014). Unlike stem‐like memory cells, which are antigen‐experienced memory cells that look like naïve cells, virtual memory cells are antigen‐inexperienced cells that display features of memory cells (i.e., naïve cells that look like memory cells without ever having encountered their cognate antigen; Chiu, Martin, Stolberg, & Chensue, 2013). These cells differentiate by cytokine‐dependent interactions (i.e., IL‐15, IL‐4; White et al., 2016) and can potentially develop from lymphopenia‐induced homeostatic proliferation (Goldrath, Bogatzki, & Bevan, 2000). Thus, the alterations in miRNA expression in old naïve CD8 cells may be potential indicators of a transition into a “virtual‐memory”‐like state. Further investigation into virtual memory cells in humans as well as the effect of homeostatic cytokines and TCR stimulation on miRNA expression in the naïve CD8 populations would provide further insight.

Similar to the miRNA findings presented here, our group has recently shown that naïve CD8 T‐cells epigenetically and transcriptionally shift toward a more differentiated state during aging (Moskowitz et al., 2017). These different processes are likely to be highly interdependent and do not follow hierarchical models. miRNA inhibition of transcription factor activity, such as FOXO1 activity, could lead to chromatin closing as well as reduced transcription of target genes. Indeed, Ucar et. al. found chromatin closing at the IL7R locus in memory CD8 T cells from older individuals (Ucar et al., 2017). There also appears to be modest, although not significant, closing in old naïve CD8 T‐cells. This chromatin closing coincides with reduced IL‐7R transcript and protein expression in old memory CD8 T‐cells compared with young (Ucar et al., 2017; our unpublished observation). Conversely, altered chromatin accessibility at loci of miRNA precursor transcripts could affect expression of the miRNAs. Indeed, we recently found the expression of miR‐181a was driven by the transcription factor YY1, which is reduced in expression during aging and YY1 motifs are highly enriched at chromatin sites less accessible in old naïve CD8 T cells (Ye et al., 2018). Thus, miRNA expression changes may be both up‐ and downstream of epigenetic changes.

Functionally, the altered miRNA network in aged naïve CD8 T‐cells predicted changes in multiple signaling pathways during aging, including those for TNFα, FOXO, and pluripotency of stem cells. Indeed, we found that old naïve CD8 T cells had transcriptional profiles similar to FOXO1 knockout cells and display features of reduced FOXO1 activity. In the field of aging, FOXO transcription factors are important regulators of longevity and stem cell homeostasis (Boehm et al., 2012; Qin & Hubbard, 2015), sharing multiple conserved targets in humans, mice, *C. elegans*, and drosophila (Webb, Kundaje, & Brunet, 2016). Likewise, FOXO1 is required for T‐cell homeostasis and T‐cell maintenance within tissues—partially via the induction of IL‐7R and other homing markers (Kerdiles et al., 2009; Ouyang, Beckett, Flavell, & Li, 2009). As we found reduced expression of IL‐7R in old naïve CD8 T‐cells correlated with lower naïve CD8 frequencies (Figure 6), it is possible that these cells have reduced homeostatic potential via FOXO1 pathway defects. This would also be consistent with a recent study characterizing human T‐cell distribution throughout tissue compartments that found significant reductions in naïve CD8 T cells in blood, lymph nodes, and spleen with age (Thome et al., 2016). Within other tissues, such as the intestinal tract, memory T‐cells predominate with very few naïve CD8 T‐cells present. As our study was limited to the investigation of peripheral CD8 T cells, it may also be of interest to investigate miRNAs and FOXO1‐related changes in these tissue‐specific naïve and memory T‐cell subsets.

Along with tissue homing and homeostasis, FOXO1 signaling also plays a vital role in the induction and maintenance of CD8 T‐cell memory in mice, with FOXO1 knockout naïve CD8 T‐cells able to generate normal antigen‐specific effector responses but unable to develop into long‐lived memory T cells (Hess Michelini, Doedens, Goldrath, & Hedrick, 2013). Knocking out FOXO1 also causes reduced viability in CD8 T cells (Delpoux et al., 2018). These cellular changes are, in turn, linked with reduced protection against secondary antigen exposure. Interestingly, older humans display normal effector responses against the zoster vaccine but have much more rapid contraction of vaccine‐specific memory cells—likely accounting for the poor efficacy of this zoster vaccine (Qi et al., 2016). In response to yellow fever vaccination, aged naïve CD8 T‐cells also demonstrate reduced effector functions (Schulz et al., 2015). Thus, FOXO1 signaling may contribute both to alterations in homeostatic proliferation and memory maintenance in human CD8 T cells during aging. Further studies on the specific role of age‐dependent miRNAs in modulating FOXO1 activity in CD8 T‐cells would provide insight into the direct relationship between miRNAs, FOXO1, and CD8 T‐cell aging.

## 4 MATERIALS AND METHODS

### 4.1 Study participants

Peripheral blood samples were obtained from individuals between 16 and 82 years of age, with no history of cancer, autoimmune disease, or diabetes mellitus, recruited in the Palo Alto area. Additional samples were purchased from Stanford Blood Center (Palo Alto, CA, USA). The study was in accordance with the Declaration of Helsinki, approved by Stanford Institutional Review Board, and all participants gave written informed consent.

### 4.2 Cell collection and subset purification

Peripheral blood mononuclear cells (PBMC) were isolated by Ficoll centrifugation. PBMCs were stained with LIVE/DEAD Fixable Aqua Dye (Thermo Fisher Scientific, Scotts Valley, CA, USA) and CD3‐Pacific Blue, CD8‐PerCpCy5.5, CD45RA‐PE‐Cy7, CD45RO‐APC, CD62L‐PE, CD127(IL‐7R)‐PE, and CCR7‐BV421 antibodies, and DUMP‐FITC (includes CD4‐FITC, CD19‐FITC, CD14‐FITC, CD56‐FITC antibodies). All antibodies were from BD Bioscience (San Jose, CA, USA) or Biolegend (San Diego, CA, USA). Live cells are sorted into naïve (DUMP^−^CD3^+^CD8^+^CD45RA^+^CD45RO^−^CD62L^+^), central memory (DUMP^−^CD3^+^CD8^+^CD45RA^−^CD45RO^+^CD62L^+^), and effector memory (DUMP^−^CD3^+^CD8^+^CD45RA^−^CD45RO^+^CD62L^−^) populations using the FACSAria (BD Bioscience) as outlined in Figure 1a.

### 4.3 miRNA profiling

miRNA was detected using Taqman Gene expression Cells‐to‐Ct Kit (Thermo Fisher Scientific) using 5,000 cells per subset as input. For multiplexing, Taqman primer/probes sets were pooled together prior to reverse transcription (RT). Following RT, pre‐amplification was performed using Taqman PreAmp Master Mix Kit (Thermo Fisher Scientific), with pooled Taqman primer/probe sets. Preamplified products were then ran on the Biomark HD (Fluidigm, San Francisco, CA, USA) for miRNA quantification. A list of miRNA Taqman primer/probes sets (Thermo Fisher Scientific) used in these studies is provided in Supporting Information Table S2. miRNA expression is relative to RNU48 expression.

### 4.4 miRNA detection by FACS

miRNA was detected by FACS using PrimeFlow RNA Assay Kit (Thermo Fisher Scientific) using PrimeFlow microRNA pretreatment buffer and type 1 microRNA probe sets for detection.

### 4.5 miRNA and mRNA pathway analyses

For the DIANA miRPATH (version 3) analysis, the six age‐dependent miRNAs from naïve CD8 T cells (hsa‐miR‐146a‐5p, hsa‐miR‐155‐5p, hsa‐miR‐181a‐5p, hsa‐miR‐7‐5p, hsa‐miR‐142‐3p, and hsa‐let‐7f‐5p) were used as input. Tarbase database (version 7), which is from experimentally validated miRNA‐mRNA interactions, was used for miRNA target selection. Gene union function was used to find enriched pathways. Pathways were selected based on the following criteria: (a) included mRNA targets for all six miRNAs; (b) a *p*‐value > 0.05; and (c) exclusion of KEGG pathways directly related to cancer, disease, or infectious pathogens. For mRNA analysis, GSEA was performed (Subramanian et al., 2005). The datasets used for the analyses described in this manuscript were obtained from dbGaP through dbGaP study accession number phs001187.v1.p1 (Moskowitz et al., 2017). Gene sets used were the hallmark gene datasets (Liberzon et al., 2015) and GSE21678 and GSE15037 for FOXO pathway analysis (Ouyang et al., 2009, 2010 ).

### 4.6 Quantitative PCR

Naïve CD8 T cells were purified from PBMCs using EasySep naïve CD8 T‐cell enrichment kit (Stem Cell Technology, Vancouver, B.C., Canada). Total RNA was extracted using RNeasy Plus Micro kit (Qiagen, Hilden, Germany) and reversed transcribed using Invitrogen Superscript VILO RT‐PCR Master Mix (Thermo Fisher Scientific). Real‐time quantitative PCR was performed with ABI Prism 7900HT Detection System (Applied Biosystems, Foster City, CA, USA) using Taqman Universal Master Mix II, no UNG (Thermo Fisher Scientific) and Taqman probe sets. Probe sets used are as follows: IL7R (Hs00902334_m1), CCR7 (Hs01013469_m1), SELL (Hs00174151_m1), TCF7 (Hs01556515_m1), and RPLP0 (Hs99999902_m1). All data are presented as relative expression normalized to RPLP0 expression, as this control gene is found to be expressed the most consistently between young and old naïve CD8 T cells.

### 4.7 Simple Western

Sorted naïve CD8 T cells were lysed with RIPA buffer (Santa Cruz Biotechnology, Santa Cruz, CA, USA) and sonicated for complete cell lysis. Lysates were centrifuged at 4°C for 10 min at 21,130 rcf, and the supernatant was collected. Protein concentration was measured using Bradford Dye (BioRad, Hercules, CA, USA) at an absorbance of 595 nm. Simple Western was performed following manufacturer's instructions at 0.2 µg/µl per sample with FOXO1 and β‐actin antibodies (Cell Signaling Technologies, Danvers, MA, USA) and ran on the Peggy Sue instrument (ProteinSimple Inc., San Jose, CA, USA).

### 4.8 In vitro inhibition assay

Cells were cultured in RPMI 1640 (Thermo Fisher Scientific) supplemented with 10% FBS (Gemini Bio Products, Sacramento, CA, USA) and penicillin‐streptomycin (Thermo Fisher Scientific) for 24 hr with FOXO1 inhibitor AS1842856 (Selleckchem, Houston, TX, USA), at indicated concentrations, or DMSO as a control. After 24 hr, expression of IL‐7R and CCR7 on naïve CD8 T cells was determined by FACS.

### 4.9 Statistical analysis

Data were analyzed using Mann–Whitney test, paired *t* test, one‐way ANOVA, or Pearson correlation as appropriate and as indicated in the specific Figure Legends. PCA was performed in r using prcomp. Statistical tests were performed using graphpad prism version 6. *p*‐Values <0.05 are considered statistically significant.

## CONFLICT OF INTEREST

All authors declare no competing interests.

## AUTHOR CONTRIBUTIONS

C.E.G. designed and performed experiments, analyzed data, and wrote the paper. M.M.C. designed and performed experiments. J.J. performed experiments and analyzed data. C.M.W. supervised the project and designed experiments. J.J.G. supervised the project, designed experiments, analyzed data, and wrote the paper.
