# Supplementary material for: Functional pathways regulated by microRNA networks in CD8 T‐cell aging
Source: Aging Cell. 2018 Nov 28;18(1):e12879. doi: 10.1111/acel.12879 (PMC6351841; doi:10.1111/acel.12879)
Supplement: Supplementary file 1 [file ACEL-18-e12879-s001.pdf]

Table S1.

	median miRNA expression					
	Young Naïve	Old Naïve	Young CM	Old CM	Young EM	Old EM
**miR-7**	2.26E-06	n.d.	1.07E-05	3.68E-06	7.43E-06	1.40E-05
**miR-9**	n.d.	n.d.	n.d.	n.d.	n.d.	n.d.
**miR-10b**	n.d.	n.d.	n.d.	1.35E-07	3.34E-07	n.d.
**miR-15a**	n.d.	n.d.	n.d.	n.d.	n.d.	n.d.
**miR-15b**	7.44E-03	5.62E-03	3.40E-02	1.77E-02	1.85E-02	1.83E-02
**miR-16**	2.06E-01	2.07E-01	9.37E-01	8.65E-01	1.08E+00	6.24E-01
**miR-17**	2.64E-02	1.09E-02	1.42E-02	1.45E-02	1.70E-02	1.35E-02
**miR-18a**	2.56E-03	2.35E-03	3.23E-03	8.76E-03	3.92E-03	5.81E-03
**miR-20a**	5.35E-01	3.32E-01	3.08E-01	3.01E-01	2.49E-01	2.88E-01
**miR-21**	3.47E-05	6.77E-04	1.48E-02	1.43E-02	6.19E-03	6.89E-03
**miR-29b**	1.26E-03	5.72E-04	2.49E-03	7.62E-04	4.11E-03	1.83E-03
**miR-92a**	1.17E+00	9.76E-01	7.40E-01	6.56E-01	7.09E-01	7.51E-01
**miR-93**	5.98E-03	1.62E-02	1.10E-02	1.54E-02	1.05E-02	2.04E-02
**miR-100**	n.d.	n.d.	n.d.	n.d.	n.d.	9.61E-01
**miR-106a**	4.53E-02	1.81E-02	2.19E-02	1.90E-02	2.35E-02	1.84E-02
**miR-106b**	2.49E-02	1.65E-02	3.10E-02	1.33E-02	2.86E-02	2.23E-02
**miR-122**	n.d.	n.d.	n.d.	n.d.	n.d.	n.d.
**miR-125a**	1.46E-05	5.85E-05	7.28E-05	2.01E-04	9.20E-05	4.15E-04
**miR-126**	4.76E-04	5.55E-04	3.51E-04	3.22E-04	3.73E-04	3.92E-04
**miR-130a**	3.57E-05	4.70E-05	7.79E-06	1.75E-06	3.09E-06	3.05E-06
**miR-142**	1.46E+01	1.03E+01	1.74E+01	1.20E+01	1.30E+01	1.21E+01
**miR-146a**	9.97E-04	3.07E-02	1.51E-01	2.61E-01	5.82E-02	2.10E-01
**miR-146b**	1.93E+00	1.67E+00	3.84E-01	7.71E-01	4.30E-01	4.39E-01
**miR-150**	1.31E+01	1.22E+01	1.49E+01	1.42E+01	1.00E+01	1.38E+01
**miR-155**	5.28E-06	3.12E-05	3.69E-04	5.08E-04	4.60E-04	7.92E-04
**miR-181a**	6.13E-04	1.89E-04	3.99E-04	5.87E-05	2.99E-04	7.11E-05
**miR-181c**	n.d.	n.d.	9.46E-07	1.10E-07	9.40E-08	5.31E-06
**miR-223**	8.75E-04	6.39E-03	2.61E-03	2.86E-03	1.11E-02	1.13E-02
**miR-301**	n.d.	1.34E-08	2.43E-07	3.68E-07	7.18E-07	5.14E-06
**miR-424**	n.d.	n.d.	n.d.	n.d.	n.d.	n.d.
**let-7a**	1.33E-02	1.01E-02	1.71E-02	1.09E-02	1.51E-02	1.40E-02
**let-7b**	4.52E-02	6.31E-02	5.95E-02	7.99E-02	4.57E-02	7.97E-02
**let-7d**	5.87E-03	3.31E-03	5.10E-03	4.53E-03	5.31E-03	4.24E-03
**let-7f**	4.07E-03	1.75E-03	4.53E-03	2.36E-03	5.63E-03	3.34E-03

Table S1. Median miRNA expression in CD8 T cell subsets by age group. n.d. = none detected

Table S2.

microRNA	Taqman cat #	Taqman Assay ID
dme-miR-7	4427975	000268
hsa-miR-9	4427975	000583
hsa-miR-10b	4427975	002218
hsa-miR-15a	4427975	000389
hsa-miR-15b	4427975	000390
hsa-miR-16	4427975	000391
hsa-miR-17	4427975	002308
hsa-miR-18a	4427975	002422
hsa-miR-20a	4427975	000580
hsa-miR-21	4427975	000397
hsa-miR-29b	4427975	000413
hsa-miR-92a	4427975	000431
hsa-miR-93	4427975	001090
hsa-miR-100	4427975	000437
hsa-miR-106a	4427975	002169
hsa-miR-106b	4427975	000442
hsa-miR-122	4427975	002245
hsa-miR-125a	4427975	002198
hsa-miR-126	4427975	002228
hsa-miR-130a	4427975	000454
hsa-miR-142	4427975	000464
hsa-miR-146a	4427975	000468
hsa-miR-146b	4427975	001097
hsa-miR-150	4427975	000473
hsa-miR-155	4427975	002623
hsa-miR-181a	4427975	000480
hsa-miR-181c	4427975	000482
hsa-miR-223	4427975	002295
hsa-miR-301	4427975	000528
hsa-miR-424	4427975	000604
hsa-let-7a	4427975	000377
hsa-let-7b	4427975	000378
hsa-let-7d	4427975	002283
hsa-let-7f	4427975	000382
RNU48	4427975	001006

Table S2. Taqman miRNA probe sets.

Figure S1.

(a) **FOXO signaling pathway (hsa04068)** **TNF signaling pathway (hsa04668)** **Pathways regulating pluripotency of stem cells (hsa04550)**

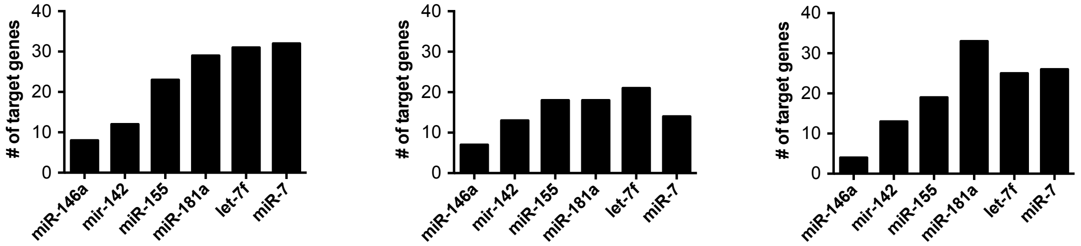

(b) **FOXO signaling pathway (hsa04068)**

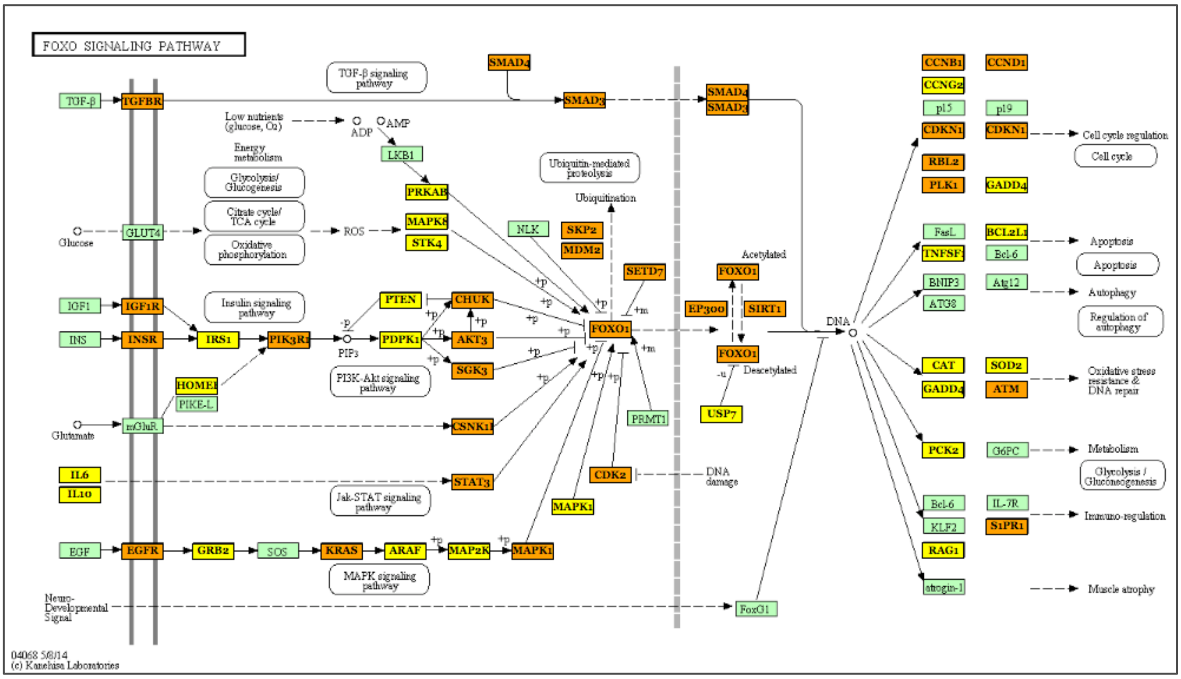

Figure S1. Targets of age-related miRNAs in the signaling pathways for FOXO, TNF and pluripotency of stem cells. (a) The number of target genes for each of the 6 age-related miRNAs from the naïve CD8 T cell analysis for the FOXO, TNF and pluripotency in stem cell signaling pathways. **(b-d)** Visual overlay of all miRNA targets onto individual KEGG pathways for the **(b)** FOXO, **(c)** TNF and **(d)** pluripotency in stem cell signaling pathways.. Light green = not targeted by any miRNA. Yellow = targeted by one miRNA. Orange = targeted by more than one miRNA.

Figure S1 continued.

(c)

TNF signaling pathway (hsa04668)

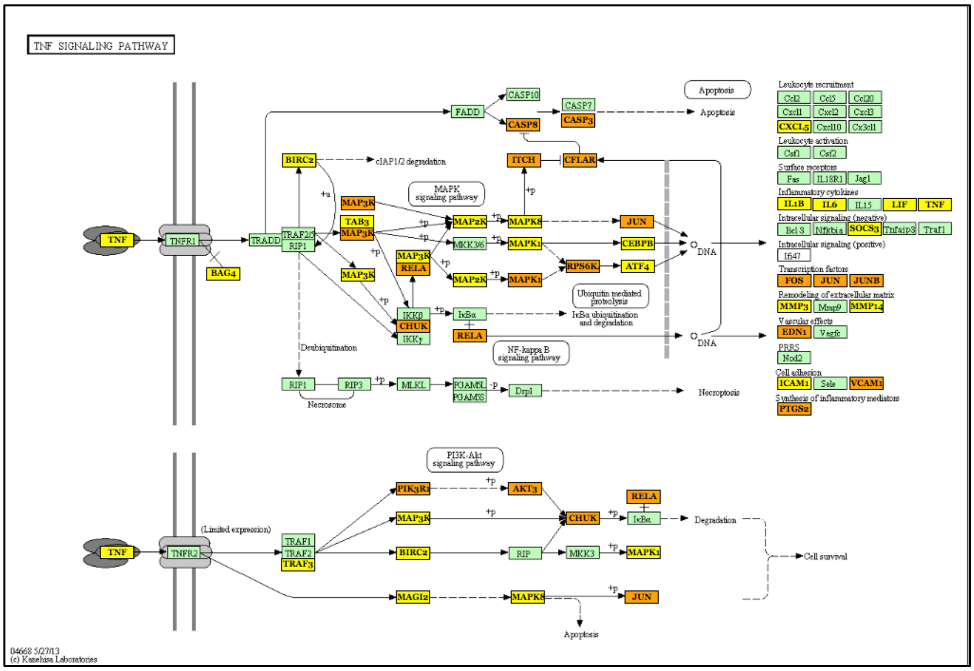

(d)

Pathways regulating pluripotency of stem cells (hsa04550)

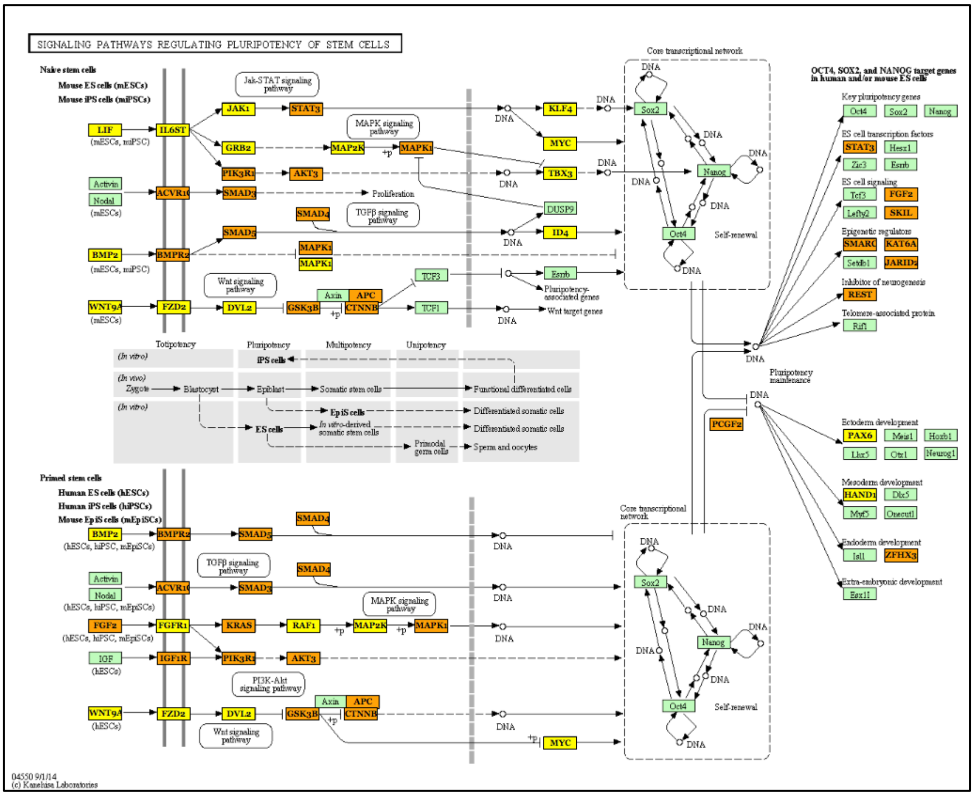

Figure S2.

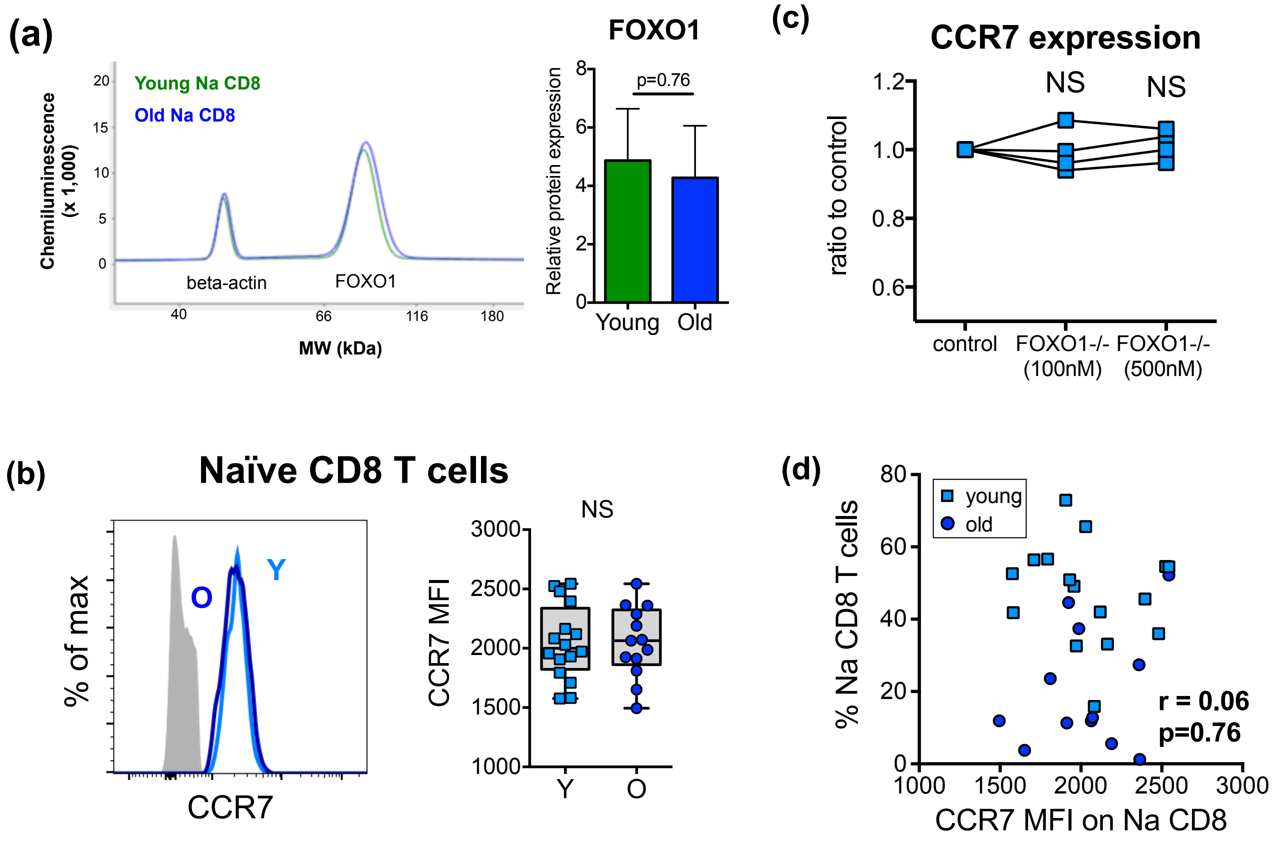

Figure S2. FOXO1 and CCR7 expression in naïve CD8 T cells. (a) FOXO1 protein expression normalized to b-actin in naïve CD8 T cells from young (n=7) and older (n=5) adults, as determined by Simple Western. **(b)** CCR7 protein expression on resting naïve CD8 T cells from young (n=16) and older (n=12) individuals; comparison by Mann-Whitney test. **(c)** CCR7 protein expression after 24 hours of FOXO1 inhibition using AS1842856 (100nM or 500nM as indicated) in young (n=4) naïve CD8 T cells. Histogram is representative of three independent experiments. P-values were determined by paired t-tests. **(d)** Pearson's correlation between baseline expression of IL-7R by naïve CD8 T cells and the frequencies of peripheral naïve CD8 T cells in young (n=16; squares) and older (n=12; circles) adults.
